# High Lateral Breakdown Voltage in Thin Channel AlGaN/GaN High Electron Mobility Transistors on AlN/Sapphire Templates

**DOI:** 10.3390/mi10100690

**Published:** 2019-10-12

**Authors:** Idriss Abid, Riad Kabouche, Catherine Bougerol, Julien Pernot, Cedric Masante, Remi Comyn, Yvon Cordier, Farid Medjdoub

**Affiliations:** 1 IEMN (Institute of Electronics, Microelectronics and Nanotechnology), Avenue Poincaré, 59650 Villeneuve d’Ascq, France; riad.kabouche@ed.univ-lille1.fr; 2 CNRS-Institut Néel, University Grenoble-Alpes, 38000 Grenoble, France; catherine.bougerol@cea.fr (C.B.); julien.pernot@neel.cnrs.fr (J.P.); cedric.masante@neel.cnrs.fr (C.M.); 3 CNRS-CRHEA, University Côte d’Azur, rue Bernard Grégory, 06560 Valbonne, France; remicomyn@gmail.com (R.C.); Yvon.Cordier@crhea.cnrs.fr (Y.C.)

**Keywords:** GaN, high-electron-mobility transistor (HEMT), ultra-wide band gap

## Abstract

In this paper, we present the fabrication and Direct Current/high voltage characterizations of AlN-based thin and thick channel AlGaN/GaN heterostructures that are regrown by molecular beam epitaxy on AlN/sapphire. A very high lateral breakdown voltage above 10 kV was observed on the thin channel structure for large contact distances. Also, the buffer assessment revealed a remarkable breakdown field of 5 MV/cm for short contact distances, which is far beyond the theoretical limit of the GaN-based material system. The potential interest of the thin channel configuration in AlN-based high electron mobility transistors is confirmed by the much lower breakdown field that is obtained on the thick channel structure. Furthermore, fabricated transistors are fully functional on both structures with low leakage current, low on-resistance, and reduced temperature dependence as measured up to 300 °C. This is attributed to the ultra-wide bandgap AlN buffer, which is extremely promising for high power, high temperature future applications.

## 1. Introduction

AlGaN/GaN high-electron-mobility transistors (HEMTs) is a promising device for high-power and high-voltage electronic applications [1,2,3,4,5,6,7,8,9]. GaN-based HEMTs are already commercially available for up to 650 V applications. However, they are currently restricted to below 1 kV mainly because of the total buffer thickness limitation with low bow and high crystal quality on large wafer diameters. SiC is another attractive wide bandgap for higher voltage but still has limited impact for the moment because of cost issues. In order to address the dynamic medium and high voltage markets beyond 1200 V while benefiting from low on-resistances, low leakage current, and low switching losses in a cost-effective way, a novel breakthrough in power electronics performance requires a new generation of materials. In this frame, the so-called ultra-wide-bandgap (UWBG) [10,11,12,13] materials such as AlN (6.2 eV), which have energy bandgaps that are larger than SiC and GaN, are very promising in enabling the next leap forward in power electronics. An AlN-based material system has a unique advantage due to its prominent spontaneous and piezoelectric polarization effects, but also its flexibility in inserting appropriate heterojunctions, thus dramatically broadening the device’s design space. Furthermore, AlN material represents the ideal back barrier for high voltage HEMT applications due to its large electrical breakdown field combined with a high thermal conductivity [14,15,16,17]. In turn, the AlN buffer can potentially not only increase the electron confinement in a transistor channel, but can also help to boost the breakdown voltage (BV), owing to its wider bandgap, while benefiting from an enhanced thermal dissipation as compared to GaN-based devices [18,19,20]. In this paper, we experimentally explore two AlGaN/GaN HEMT structures using a sub-10 nm thin and 240 nm thick GaN channel that is grown on AlN/sapphire templates.

## 2. Material Description and Device Fabrication 

**Figure 1:**
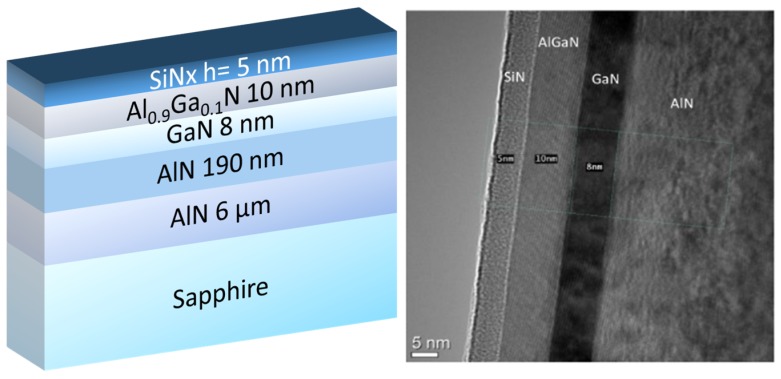
Schematic cross section of the thin channel structure (left) and high resolution Transmission Electron Microscopy picture of the active layers grown by molecular beam epitaxy (MBE) (right).

**Figure 2:**
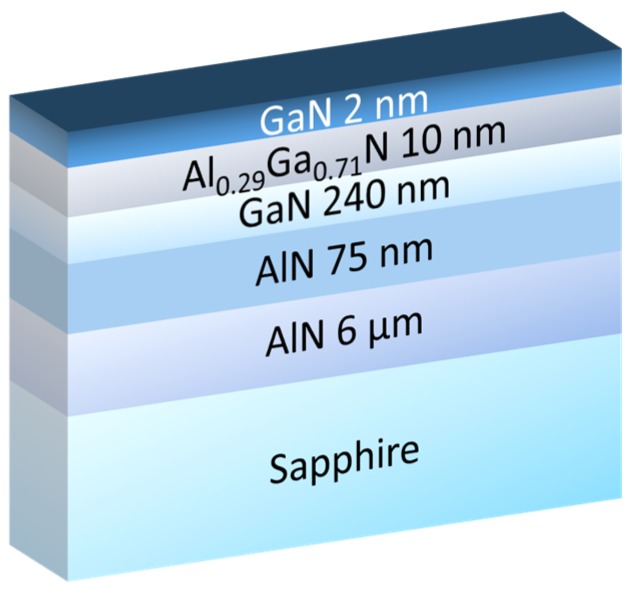
Schematic cross section of the thick channel structure.

Figure 1 shows a schematic cross section of the AlN-based heterostructures grown by ammonia molecular beam epitaxy (MBE). The first structure, referred to as the thin channel structure, includes a 190 nm AlN buffer that was regrown on a 6 µm thick AlN-on-sapphire commercial template followed by a 8 nm thin GaN channel, a 10 nm AlGaN barrier layer with a high aluminum content (90%), and a 5 nm in-situ SiN cap layer. The growths were performed in a Riber Compact 21T MBE system that was equipped with 80 cc crucible effusion cells that were designed to supply a uniform flux of group III elements on 50 mm wafers [21]. After outgassing under high vacuum at 500 °C, the AlN-on-sapphire templates were annealed under NH_3_ (flow rate 200 sccm) at 850 °C prior to the growth of AlN buffer using the same conditions at a growth rate of 100 nm/h. Then, the temperature was reduced to 780 °C in order to grow the rest of the structure, starting with the GaN channel. Based on previous calibration samples, the growth rates of GaN and AlN were then adjusted to produce AlGaN barriers with the desired Al content. While the structure with a 240 nm GaN channel and 29% Al barrier was capped with a thin GaN layer in quite a standard procedure, which produced high performance Radio Frequency HEMTs on silicon [22], the structure with 90% Al barrier was capped with a 5 nm SiN layer in-situ grown at 700 °C using a silicon sublimation source (SUSI) from MBE Komponenten (Weil der Stadt, Germany). The crystal quality of AlN was assessed by X-ray diffraction (XRD) using omega scans. Rocking curves around the symmetric (002) reflections are mainly sensitive to screw type threading dislocations and asymmetric (101) reflections are sensitive to any type of threading dislocations, edge, mixed, and screw type. The full widths at half maximum (FWHM) of AlN (002) and AlN (101) reflection peaks were estimated below 350 arcsec and 500 arcsec, respectively, for both samples, indicating a mean threading dislocation density in the range of fewer than 10^8^/cm². On the other hand, the signal of GaN reflections were too weak for the correct determination of (002) and (302) peak widths for the 8 nm channel, whereas they were evaluated at 450 arcsec and 1940 arcsec for the 240 nm GaN layer, indicating a mean threading dislocation density in the range of fewer than 10^9^/cm². Interestingly, such values are in a similar range as 1,7 µm thick GaN layers were developed to produce high performance HEMTs on silicon [22,23]. The 2DEG properties that were obtained by the Hall Effect measurements show a sheet carrier density of 1.9 × 10^13^ cm^−2^ and an electron mobility of 340 cm²/V·s. The rather low mobility can be attributed to the thin channel and/or the high Al content into the barrier layer, which can still be optimized, with large room for improvement. A high resolution Transmission Electron Microscopy picture of the active layers is also shown in Figure 1. Sharp interfaces and high material quality are observed. The second structure (Figure 2), which was grown on a similar template, consists of a 240 nm thick GaN channel, a 10 nm AlGaN barrier (29% Al), and is capped with a 2 nm GaN layer. The 2DEG properties show a charge density of 1.1 × 10^13^ cm^−2^ and a higher electron mobility of 1500 cm²/V·s.

For the two structures, a Ti/Al/Ni/Au metal stack was used to form the ohmic contacts on top of the barrier layers by fully etching the cap layers, using a Fluorine-based plasma for the SiN cap layer and a Chlorine-based plasma for the GaN cap layer. A rapid thermal annealing was applied at 750 °C and 875 °C on the thick and thin channel structures, respectively. Contact resistances of about 0.6 Ω·mm were obtained. Then, the device isolation was achieved by nitrogen ion implantation. Ni/Au stack was used as a gate metal and was deposited on top of the cap layers. 

## 3. Results and Discussion

**Figure 3:**
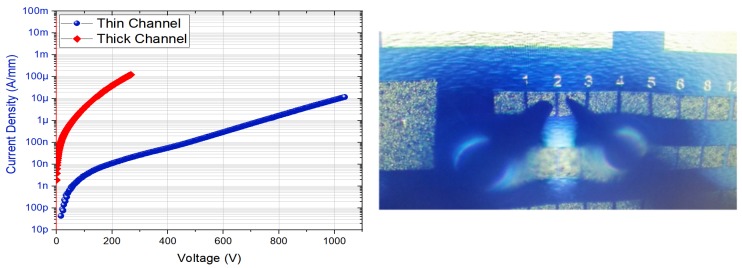
Lateral breakdown voltage at Room Temperature on isolated contacts with a 2 µm distance of thin and thick channel structures. The probes that were emerged in a Fluorinert solution on top of the contacts are also shown.

Figure 3 shows typical lateral breakdown measurements performed on isolated contacts with a 2 µm spacing for both structures while the substrate is floating. The probes are emerged in a Fluorinert solution in order to avoid electrical arcing in air under high voltage. The thin channel structure shows a lateral BV that is slightly higher than 1000 V, which corresponds to a remarkable breakdown field above 5 MV/cm. It can be pointed out that this value is far beyond that of a GaN-based material system [24]. To the best of our knowledge, this is the first high voltage demonstration on AlN-based HEMTs using a thin GaN channel. The lateral BV was measured on the thick channel structure in the same way, resulting in 270 V for the same contact distance of 2 µm (<2 MV/cm). As expected, BV is dominated by the thick GaN channel in this case and is thus similar to the best conventional GaN-based HEMTs. However, it appears that the breakdown mechanism may not be a limitation when using a sub-10 nm thickness, enabling it to benefit from the AlN bandgap for short contact distances (i.e., under high electric field). 

**Figure 4:**
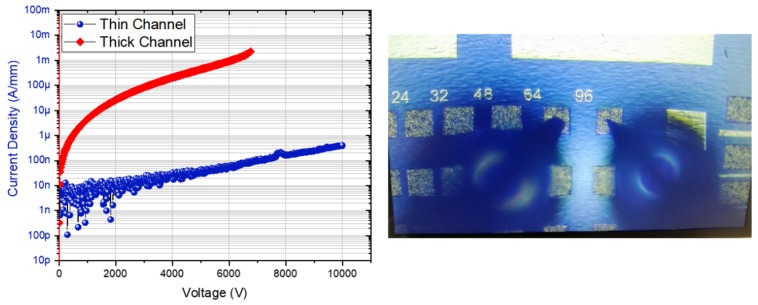
Lateral breakdown voltage at RT on isolated contacts with a 96 µm distance of thin and thick channel structures. The probes that were emerged in a Fluorinert solution on top of the contacts are also shown.

**Figure 5:**
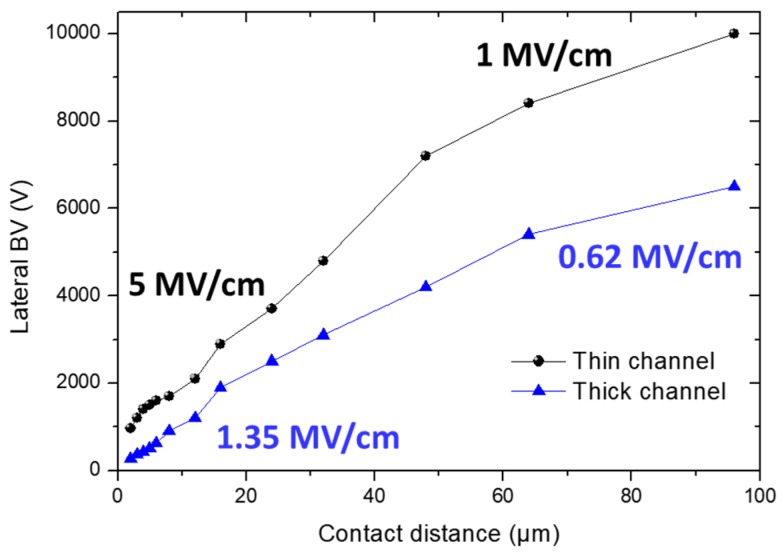
Lateral breakdown voltage at RT on isolated contacts with a 96 µm distance of thin and thick channel structures. The probes that were in a Fluorinert solution on top of the contacts are also shown.

Figure 4 shows the lateral breakdown measurement on isolated contacts with a 96 µm spacing using a Keysight B1505A with N1268A Ultra High Voltage Expander. A significant lateral breakdown up to 10 kV (limit of the set-up) is achieved with a leakage current of 300 nA/mm on the thin channel structure. The very high blocking voltage and the low leakage current show that the heterostructure does not suffer from any parasitic conduction as full depletion down to the sapphire substrate may occur. The thick channel structure yields a value below 7000 V. On top of the much lower BV, we can also notice a higher leakage level compared to the thin channel structure. Figure 5 represents the lateral BV for various contact distances, confirming a systematically lower breakdown field for the thick channel structure. The lower breakdown field for the larger contact distances can be attributed to the regrown interface states that are activated under very high electric field. Considering that the AlN templates and the MBE regrowth are similar, it can be stated that the sub-10 nm channel thickness is beneficial for high voltage operation in this material system. 

**Figure 6:**
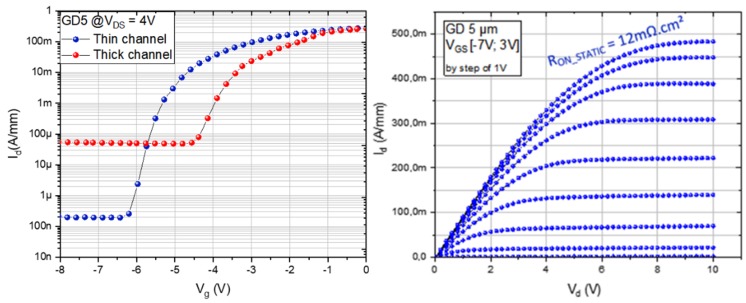
Transfer characteristics (left) for thin and thick channel structures and output characteristics (right) of the thin channel structure at RT.

DC characteristics of both structures show fully functional devices, as seen in Figure 6 for both structures. For the thin channel structure, the off-state leakage current at V_DS_ = 4 V is around 200 nA/mm and the static on-resistance R_ON_ scales are expected with the gate-drain distance to reach values below 15 mΩ·cm^2^ for a 5 µm distance. However, despite fully functional transistors with low leakage current and low on-resistance, a rather limited BV on transistors of about 600 V was measured, which is attributed to the non-optimized SiN passivation layer. On the other hand, in agreement with the buffer characteristics, more than two orders of magnitude higher off-state leakage current is observed on thick channel transistors.

**Figure 7:**
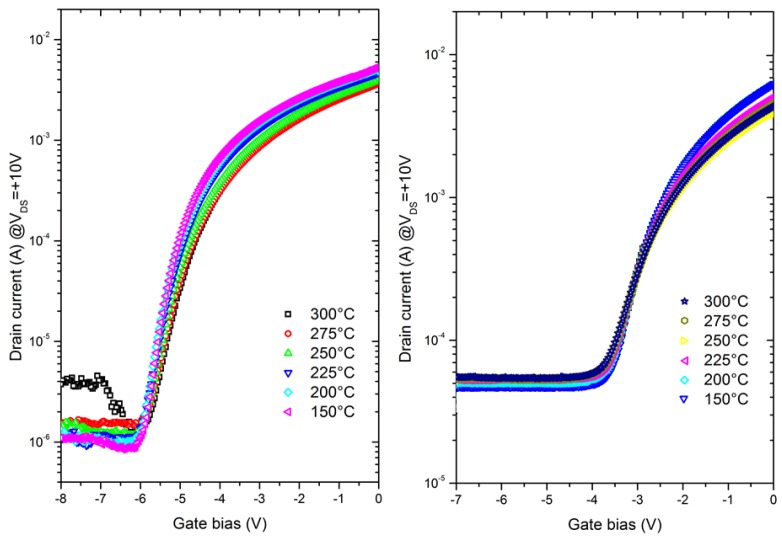
Transfer characteristics at V_DS_ = +10 V as a function of temperature (from 150 °C to 300 °C) of the thin channel structure (left) and the thick channel structure (right).

Figure 7 depicts the transfer characteristics at various temperatures for the thin and thick channel structures at V_DS_ = 10 V. A low variation of the off-state leakage current and the threshold voltage (V_TH_) is observed up to 300 °C even though the thick channel structure delivers a higher leakage current level. It can be stressed that at such a high temperature, AlGaN/GaN HEMTs using standard (Al)GaN-based buffer layers generally show V_TH_ shift and especially significant leakage current increase due to a higher charge conductivity within the buffer layers. Therefore, it appears that the use of AlN material as a buffer may enable it to push the temperature limitation of GaN-based transistors. Furthermore, as expected, the on-state current density drops with the temperature increase due to the decrease of the electron mobility, which is induced by increasing phonon scattering [25]. However, the current density degradation is lower in the case of the thin channel. This indicates that the channel temperature for the thin channel devices is lower under the same bias conditions. This effect is probably due to the lower dislocation density at the GaN/AlN interface in the case of the thin layer, in turn reflecting the benefit of the high AlN thermal conductivity [26,27]. Further experiments such as Infrared/Raman thermography would be needed in order to fully confirm the thermal stability improvement.

## 4. Conclusions

This work presents the fabrication and characterization of thin and thick channel AlGaN/GaN HEMTs grown on AlN/sapphire templates. Lateral buffer breakdown voltage assessment of the thin channel reveals a remarkable breakdown field of 5 MV/cm for short contact distances, which is far beyond the theoretical limits of a GaN-based material system. Furthermore, fabricated transistors are fully functional with low leakage current and low on-resistance. The use of a sub-10 nm ultrathin GaN channel may not be a limiting factor for the breakdown mechanism of transistors. Temperature measurements up to 300 °C show that the AlN buffer enables increased temperature stability of GaN-based transistors. When combined with a thin channel, the related transistors may deliver lower channel temperatures as compared to devices with thicker channels. Proper passivation and associated processing will allow us to take advantage of the properties offered by AlN-based devices, providing both low resistances and high voltages well above 1 kV.
